# The Impact of the COVID-19 Pandemic on the Cognition of People with Dementia

**DOI:** 10.3390/ijerph18084285

**Published:** 2021-04-18

**Authors:** Giacomo Tondo, Barbara Sarasso, Paola Serra, Fabiana Tesser, Cristoforo Comi

**Affiliations:** 1 Neurology Unit, S. Andrea Hospital, Department of Translational Medicine, University of Piemonte Orientale, Corso Abbiate 21, 13100 Vercelli, Italy; giacomo.tondo85@gmail.com (G.T.); barbara.sarasso@aslvc.piemonte.it (B.S.); paola.serra@aslvc.piemonte.it (P.S.); fabiana.tesser@aslvc.piemonte.it (F.T.); 2 School of Psychology, Vita-Salute San Raffaele University, 20132 Milan, Italy; 3 Interdisciplinary Research Center of Autoimmune Diseases (IRCAD), University of Piemonte Orientale, 28100 Novara, Italy

**Keywords:** coronavirus disease, cognitive decline, quarantine

## Abstract

(1) Background: To limit the COVID-19 outbreak, the Italian government implemented social restrictions that may have had psychological and cognitive repercussions on people with dementia. We aimed to analyze cognitive decline during the pandemic year in people evaluated in a memory clinic in northern Italy, the epicenter of COVID-19 spread. (2) Methods: A single-center retrospective study was carried out, including individuals with annual follow-up evaluated in three different years (2020-GROUP, 2019-GROUP, 2018-GROUP). We performed an intergroup comparison of cognitive decline over a one-year follow-up, and an intragroup comparison in the 2020-GROUP to analyze the five-year cognitive decline trajectory, as measured by the MMSE. (3) Results: The 2020-GROUP showed a significant loss of MMSE points per year in the considered follow-up period compared with the 2019-GROUP and 2018-GROUP (*p* = 0.021). Demographics, clinical features, and the other analyzed variables, including rate of diagnosis, therapy, and comorbidities, did not significantly differ between groups. The five-year cognitive decline trajectory confirmed a significant worsening of cognitive decline between 2019 and 2020 (*p* < 0.001), while the decrease in MMSE scores was not statistically significant between previous time points. (4) Conclusions: COVID-19 pandemic measures have induced a significant worsening of cognitive decline in people with dementia, needing more careful assistance to minimize the adverse effects of social isolation in case of future lockdowns.

## 1. Introduction

The outbreak of the novel coronavirus SARS-CoV-2, causing an acute respiratory illness with heterogeneous systemic symptoms (COVID-19), exploded in Europe with the first infected patients officially diagnosed in Italy in February 2020. One year later, on February 1st 2021, the total diagnosed cases in Italy amounted to about 2.5 million individuals [1]. To limit the spread of COVID-19, the Italian government implemented a set of increasingly severe restrictions, such as social isolation and limitation in individual movements. Due to the increased pressure on hospitals related to COVID-19 diffusion, regional governments elaborated the temporary closure of outpatient clinics, maintaining only essential services. The healthcare system’s extreme difficulties drastically changed patient management in both clinical and surgical settings, especially in northern Italy, where the impact of the COVID-19 pandemic was particularly crushing [2,3,4]. For patients with dementia and their relatives, social and familial repercussions of COVID-19, peaking in the period of self-isolation, entailed an increased risk of adverse outcomes, associated with more challenging access to the healthcare system, reduction of non-urgent clinical activities, fear of contracting the virus, and concerns of not receiving adequate hospital care. It is undebatable that the frail aged population suffered higher distress [5]. Older adults with dementia have a high risk of contracting COVID-19 and, once infected, have a high risk of disease-related morbidity and mortality [6]. Dementia represents a major risk of adverse outcomes during COVID-19 infection [7], and patients with cognitive impairment show more severe clinical manifestations and a higher mortality rate than people without cognitive impairment [8,9].

The aged population is particularly prone to distress linked to the effects of the disease and the negative consequences of the social restriction rules. Social isolation and compulsory quarantine during the COVID-19 pandemic were associated with an increased rate of psychiatric symptoms including stress, anxiety, insomnia, and depression in the general population [10,11,12], in families [13], and in specific overexposed categories, such as healthcare workers [14,15], students [16], and caregivers of people with dementia [17]. As expected, in patients with dementia, quarantine induced the worsening of preexisting neuropsychiatric disturbances or the rise of new psychological and behavioral symptoms, with additional distress for caregivers [18]. Measures limiting agitation in people with dementia can exasperate neuropsychiatric symptomatology, and the worsening of behavioral disturbances further complicates management in this population, possibly resulting in chronic, more challenging disorders [19]. Lastly, but not less important, people with dementia often carry multiple comorbidities, take several medications, and are dependent on daily living activities, which require the implementation of caregiver support and medical assistance [20].

To date, the trajectory of cognitive decline of people with dementia during the COVID-19 pandemic has not yet been analyzed. In the current study, we collected data in a sample of patients with cognitive impairment who visited the Centre for Dementia and Cognitive Disorders (CDCD) in 2020 in Vercelli, Piedmont, one of the epicenter regions of COVID-19 spread in northern Italy. To evaluate the impact of the COVID-19 pandemic on people with dementia, we analyzed cognitive decline during the year of the outbreak, and we compared cognitive performances between groups of patients visited in previous years.

## 2. Materials and Methods

### 2.1. Subjects

**Figure 1:**
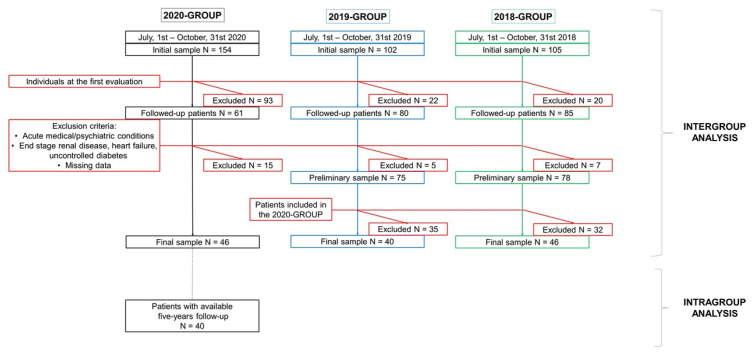
Sample selection flow chart for individuals with cognitive impairment evaluated at the Centre for Dementia and Cognitive Disorders in Vercelli, over the period July–October 2020, 2019, and 2018. In the 2020-GROUP, to perform a further intergroup analysis, data collection was extended to charts of individuals with annual follow-up in the period 2016–2020 to evaluate the five-year trajectory of cognitive decline.

We performed a single-center retrospective study at the CDCD of the Neurology Unit, Sant’Andrea Hospital in Vercelli, Piedmont, Italy. The main group was created by screening the clinical records of people evaluated from July, 1st to October, 31st 2020 (2020-GROUP). During these four months, regular activity of the CDCD was temporarily restored by the regional government, following the end of the Italian “first pandemic wave”, with a decrease of COVID-19 cases and consequently reduced pressure on the national health system [1]. A total of 154 patients were evaluated during the target period. In the current study, we selected only participants with at least one previous evaluation (T0) before the target period (T1). We excluded patients at their first evaluation (N = 93) and patients with missing data (N = 5). Individuals with acute medical or psychiatric conditions and patients with end-stage renal disease, heart failure, or uncontrolled diabetes were also excluded (N = 10). For comparison, we used the same sample selection strategy to include consecutive subjects who had a visit at the same CDCD between July and October 2019 (2019-GROUP) and between July and October 2018 (2018-GROUP), excluding also individuals already included in the 2020-GROUP. Final samples included N = 46 individuals evaluated between 2019 and 2020, N = 40 individuals evaluated between 2018 and 2019, and N = 46 individuals evaluated between 2017 and 2018. Figure 1 details the sample selection process. The study was conducted in strict accordance with the principles of the Declaration of Helsinki. Prospective informed consent and ethical review and approval were waived due to the retrospective nature of the study and the use of pseudonymized data. 

### 2.2. Data Collection

We reviewed subjects’ charts with the complete medical records, including general medical status, sex, age, education, and comorbidities. During each visit, all included subjects were assessed with a standard neurological evaluation performed by a neurologist and a psychologist with cognitive impairment and dementia expertise. All diagnoses were established according to international consensus criteria [21,22,23,24,25]. Each participant had a mini mental state exam (MMSE) [26] as a global mental status screening tool, available both at the T0 and at the T1 visit. To provide a measure of cognitive decline in the three groups, we estimated the index of progression (IP) calculated by the formula: follow-up MMSE −baseline MMSE/years of follow-up, previously used to compare the progression of cognitive deterioration in different groups of individuals with cognitive impairment [27]. 

We performed two main comparisons: (a) an intergroup comparison over a one-year follow-up, between 2020, 2019, and 2018-GROUP; (b) an intragroup analysis of the 2020-GROUP, collecting all available data since the year 2016, comparing the annual change of cognitive performance over a longer follow-up period (five-year trajectory, see Figure 1). 

### 2.3. Statistical Analysis

Data analysis was conducted with IBM SPSS Statistics for Windows, Version 25.0 (IBM Corp., Armonk, NY, USA). The data were tested for normality with Shapiro–Wilk statistical tests. Quantitative variables were described using mean and standard deviation, while qualitative variables were expressed as a number and percentage. Differences in demographic, clinical, and cognitive features were assessed using the Kruskal–Wallis test e for continuous and χ^2^ tests for categorical variables. Differences in annual changes in MMSE in the 2020-GROUP over the period 2016–2020 were evaluated using the repeated measures analysis of variance (rm-ANOVAs). The Greenhouse–Geisser correction was used to correct for non-sphericity. Bonferroni’s post hoc correction was applied for further analyses. A *p* value < 0.05 was considered significant for all analyses.

## 3. Results

### 3.1. Intergroup Analysis

**Figure 2:**
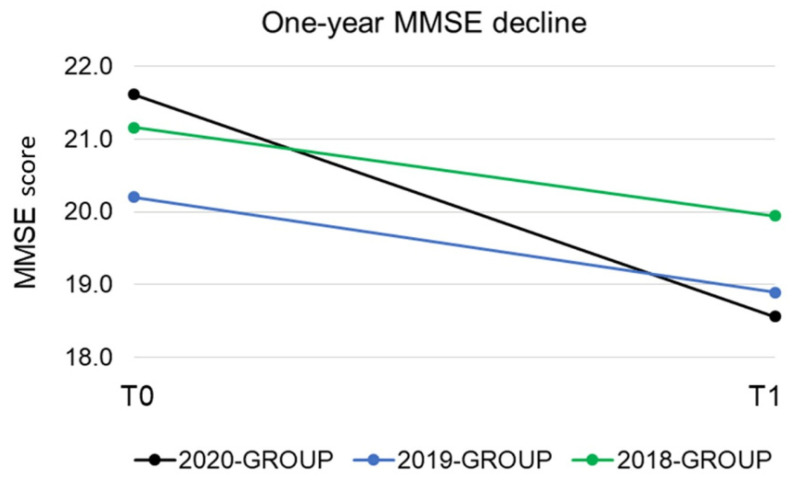
Annual mini mental state examination decline in the 2020-GROUP (black line), 2019-GROUP (blue line), and 2018-GROUP (green line). The 2020-GROUP showed a significantly higher loss of MMSE points per year, as calculated by the index of progression, than the 2019-GROUP and 2020-GROUP. MMSE: mini mental state examination.

The whole sample consisted of N = 132 subjects, grouped in the 2020-GROUP (N = 46), 2019-GROUP (N = 40), and the 2018-GROUP (N = 46). None of the subjects included in the 2020-GROUP had contracted COVID-19. Table 1 details the main demographic, clinical, and cognitive features for the three groups. Age, sex, and educational level did not differ between the three groups. Mean disease duration, defined as the duration since onset of cognitive impairment, was very similar among groups (4.33 ± 2.24 years in the 2020-GROUP, 4.10 ± 1.98 years in the 2019-GROUP, and 3.96 ± 1.80 years in the 2018-GROUP). MMSE corrected scores at T0 and T1 did not differ across groups. The 2020-GROUP showed a significantly lower IP (IP = −3.25) than the 2019-GROUP (IP = −1.39) and 2018-GROUP (IP = −1.33), indicating a significantly higher loss of MMSE points per year in the considered follow-up period for patients included in the 2020-GROUP (*p =* 0.021) (Figure 2).

**Figure 3:**
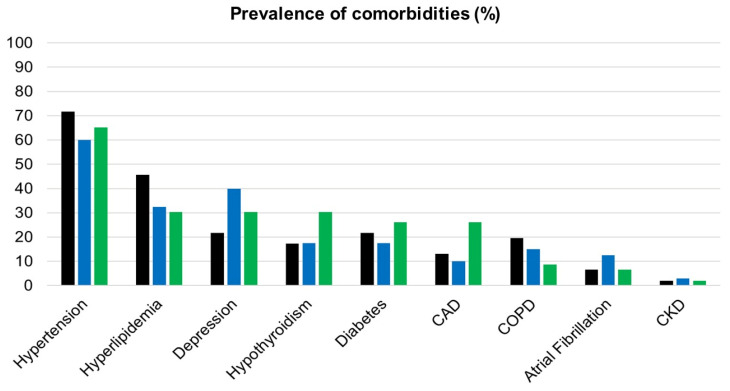
Different prevalence of comorbidities in the three groups: 2020-GROUP (black bars), 2019-GROUP (blue bars), and 2018-GROUP (green bars). CAD: coronary artery disease; COPD: chronic obstructive pulmonary disease; CKD: chronic kidney disease. Differences between groups were assessed with Chi-squared tests. None of the considered variables showed a statistically significant difference in the comparison between groups.

To exclude the effect of other variables on the more severe cognitive decline observed in the 2020-GROUP, we investigated whether there were significant differences in the frequency of different dementia subtypes (considering probable Alzheimer’s disease, vascular dementia, frontotemporal dementia, dementia with Lewy bodies, and mild cognitive impairment) and in the use of cholinesterase inhibitors and/or memantine. The most frequent diagnosis was Alzheimer’s disease in the three groups, representing 46% of the sample in 2020-GROUP, 52% in the 2019-GROUP, and 57% in the 2018-GROUP. No differences between groups were found regarding the frequency of each diagnosis (see Figure 3). The most used cholinesterase inhibitor was donepezil in all three groups, followed by rivastigmine, and by the association of donepezil plus memantine, with similar distribution among groups.

Since comorbidities in people with dementia may negatively affect cognitive performance, we investigated whether the frequency of comorbidities, including atrial fibrillation, coronary artery disease, chronic obstructive pulmonary disease, depression, diabetes, hyperlipemia, hypertension, and hypothyroidism, differed between the three groups. The majority of subjects in the three groups suffered from hypertension, followed by hyperlipemia. Depression was slightly less prevalent in the 2020-GROUP, affecting 10 patients (22%), in comparison with 16 patients in the 2019-GROUP (40%) and 14 (30%) in the 2018-GROUP. Such differences were not statistically significant (*p* = 0.185). The three groups did not show any statistically significant differences in all the other considered comorbidities (results are represented in Figure 3).

### 3.2. Intragroup Analysis

**Figure 4:**
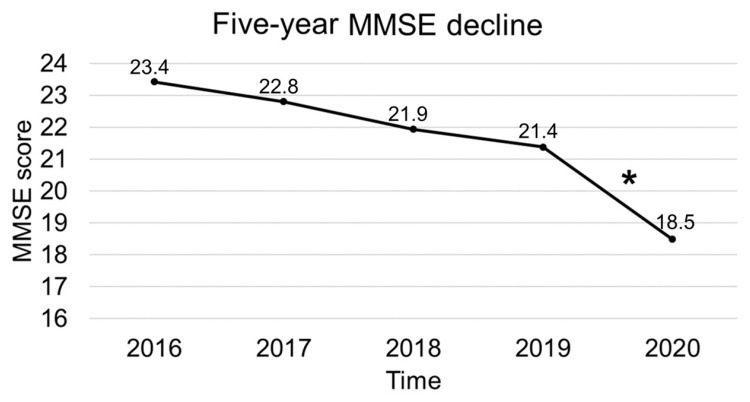
Five-year mini mental state examination decline in N = 40 patients of the 2020-GROUP evaluated annually from 2016 to 2020. MMSE corrected scores significantly decreased between 2019 and 2020 (asterisk indicates the significant difference, *p* < 0.05). MMSE: mini mental state examination.

In the intragroup analysis, we collected all data from patients evaluated at least once per year from 2016 to 2020 (N = 40). The rm-ANOVAs with a Greenhouse–Geisser correction, conducted to compare scores on the MMSE over time, showed that mean MMSE corrected scores differed significantly between time points (F(2.197, 85.686) = 41.943, *p* < 0.001). Post hoc tests using Bonferroni correction showed that MMSE corrected scores significantly decreased between 2019 and 2020 (*p* < 0.001), while the decrease in MMSE scores was not statistically significant between previous time points (Figure 4).

## 4. Discussion

The present study aimed to investigate cognitive decline during the COVID-19 pandemic in people with dementia, evaluated in a northern Italy CDCD immediately after the first Italian lockdown. Compared with groups of patients previously evaluated in the same CDCD and before the COVID-19 outbreak, elderly people with cognitive impairment who visited in 2020 showed a significantly greater loss of MMSE points per year. When analyzing the cognitive decline trajectory over a five-year follow-up, a significant effect of time between 2019 and 2020 evaluations was found, suggesting that the lockdown may have elicited a significant reduction in MMSE score. 

Evidence from the past few months suggests a link between COVID-19 and cognitive decline. COVID-19 infections may lead to acute neurological and psychiatric complications [28]. Cerebrovascular events, altered mental status, and delirium represent common manifestations, which can worsen a preexisting cognitive deficit [29]. The precise mechanisms by which the virus damages the brain are not fully elucidated yet, but the virus seems to have a particular tropism for the nervous system, as evidenced by the lack of taste and smell as one of the most frequent symptoms [30]. Recently, chronic and long-term neurological sequelae have been hypothesized, including neurocognitive symptoms [31]. While the reports on cognitive manifestations of COVID-19 infection are growing, data on the effects of lockdown and the COVID-19 pandemic-related measures on people with dementia are still sparse. 

Several aspects of the impact of COVID-19 were analyzed and reported on from different countries in elderly people with and without cognitive impairment, but data are mainly based on surveys and telephone interviews. The pandemic produced psychological disorders, including depression, anxiety, and loneliness in elderly people with cognitive impairment living at home, suffering from a decrease in social activities [32]. Isolation and health needs have emerged as crucial issues for family experiences [33]. The disruption in routine activities has produced a significant overall decline and a reduced autonomy in daily living activities in care recipients, as reported by their caregivers [34,35]. Coherently, worries of faster cognitive decline have been reported both in patients and in caregivers, and also in cognitively normal individuals, all experiencing increased psychological symptoms [36]. In fact, the most consistent finding reported during the pandemic period regards a higher rate of neuropsychiatric symptoms due to social isolation in people with dementia [19]. An increased burden of behavioral and psychological disturbances has been reported in about 60% of persons with dementia living in the community [18]. Consequently, caregivers of people with dementia also experienced an increased rate of psychological symptoms, including anxiety and depression [17,37]. In addition to neuropsychiatric disorders, a high rate of delirium episodes and falls has been reported in those with cognitive impairment [38], and these events are known to be associated with the worsening of cognitive performance [39,40,41]. The healthcare system struggled to copy with the health needs of people with dementia living in the community and in a protected environment. A higher prevalence of adverse events, i.e., injury resulting from medical care or failure to provide care, has been reported in a large cohort including people living in nursing homes, covering the whole Italian territory, with increases in the use of antipsychotic and physical restraints as the factors associated with the occurrence of adverse effects [42]. Overall, an increased rate of prescription of antipsychotics has been registered during the pandemic [43]. The presence of behavioral problems, a greater burden of psychotic symptoms, and the use of antipsychotics or antidepressant may further facilitate cognitive decline in people with dementia [44,45]. 

To study the impact of the pandemic on people with dementia, we created three groups, including patients from three different years, but going to the same CDCD, in the same period of the year (namely, July–October), and evaluated them two times in a one-year follow-up, which was identical between groups. The groups were also well matched with regard to demographic variables, including sex, age, and educational level. No differences were found regarding anticholinesterase therapy between groups. However, several other factors could have influenced cognitive performances in the 2020-GROUP. In all three groups, the inclusion of patients with different subtypes of dementia and disease durations needed to be considered. The natural history of cognitive decline in AD patients may differ from that of other types of dementia [46,47,48], and MMSE decrease may vary based on the disease phase [49,50]. Nonetheless, we did not find statistically significant differences in mean disease duration and frequency of dementia subtypes when comparing the three groups. Lastly, comorbidities in older people may affect the rate of cognitive function. Atrial fibrillation, coronary artery disease, chronic obstructive pulmonary disease, depression, diabetes, hyperlipemia, hypertension, and hypothyroidism are known to modulate cognitive function in the elderly and in patients with dementia [51,52,53,54,55,56,57]. All these comorbidities were equally represented among groups, therefore we can reasonably exclude that the effect of concurrent medical conditions drove the cognitive decline in the 2020-GROUP.

Our data do not allow us to identify the precise cause of the severe cognitive decline detected in our population. Nevertheless, we can hypothesize a crucial role for the pandemic-related restrictive measures and their consequences, including social isolation, increasing difficulties in coping with the limitations of daily living activities, the break of usual routines, the psychological burden on caregivers, and the temporary closure of the reference CDCD. These aspects may have exacerbated cognitive decline in particularly frail people, as suggested by the finding of a greater cognitive decline after considering all the possible confounding variables. Some criticisms in our sample selection strategy and methods need to be specified. Worries of being infected by approaching the outpatient clinic might have limited the access to our CDCD for the most severe cases. This aspect could partially be responsible for the difference in cognitive decline observed between groups enrolled in different years. However, a significant decline in MMSE score between 2019 and 2020 was observed in the intragroup analysis. This result confirms that the 2020-GROUP patients experienced, over the pandemic period, a significant deterioration that they had never expressed in previous years. Unfortunately, due to data unavailability, we could not quantify the mortality rate in our selection. However, we can assume higher mortality in our target population, as previously reported [58]. As a consequence, real data on cognitive decline in people with dementia might be even more severe than the presented data due to the lack of information about most critically affected patients, showing a higher rate of mortality than less frail ones [59]. 

To assess cognitive decline in our patients, we considered the annual decrease in MMSE. MMSE is the most widely used global cognitive status screening scale, showing undebatable advantages, including validity for screening demented, aged people and good interrater reliability, but also some caveats, especially in patients with high premorbid intelligence and high education, which may lead to false negatives [60]. In addition, social isolation can affect MMSE performances [61], possibly due to the lack of spatial and temporal benchmarks. In our study, the 2020-GROUP included elderly patients (mean age 78.78 ± 8.82 years) with a mild-low educational level (mean education 6.96 ± 3.63 years) and a mild-to-moderate cognitive decline (mean baseline MMSE score 21.61 ± 4.75). We can assume that MMSE in this sample reliably depicts the worsening of global cognition in people with dementia referring to a CDCD. These results should represent a wake-up call for the healthcare system, needing a change in the management of cognitively impaired patients.

In the current study, we did not assess the burden of neuropsychiatric symptoms. Moreover, when assessing mood disorders, we only considered depression. The lack of a semi-quantitative measurement of depression may represent a further limitation linked to the retrospective analysis we conducted. However, the rate of depression did not differ significantly in the three groups, having been shown to be slightly less prevalent in the 2020-GROUP compared with the other groups, and it was similar to that reported in the literature in demented people, ranging from 22% to 40% [62]. A further prospective investigation is needed to study the higher presence of mood and behavioral disturbances and their relationship with cognitive decline in frail populations exposed to the burden of the pandemic. 

Our results are also limited by the small sample size and by the single measure used to evaluate cognitive decline, which is MMSE. However, they crucially provide novel insights into challenges faced by people with dementia during the COVID-19 pandemic, underlying the need for constant assistance for patients and caregivers to mitigate the risk of worsening cognitive and psychological outcomes.

## 5. Conclusions

This study sheds light on the difficulties of people with dementia during the COVID-19 pandemic. 

COVID-19 pandemic-related measures, including changes in routine activities, restrictions in individual movements, and the impediment to access to the hospitals and follow-up care, may have impacted on both psychological and cognitive levels. 

Our results confirm that people with dementia suffered from a significant worsening of cognitive impairment between 2019 and 2020, which seemed unrelated to the diagnosis, disease duration, and comorbidities. These findings underline the criticisms that the healthcare system will face: the need to minimize the adverse effects of social isolation, especially in particularly frail people who would benefit from closer assistance in case of a future lockdown.

## Figures and Tables

**Table 1:** Subject demographic, clinical, and cognitive characteristics.

	2020-GROUP N = 46	2019-GROUP N = 40	2018-GROUP N = 46	*p* Value
Age	78.78 ± 8.82	79.58 ± 6.20	79.30 ± 6.21	0.919
Sex (f/m)	32/14	25/15	28/18	0.654
Education in years	6.96 ± 3.63	6.25 ± 2.69	6.43 ± 3.59	0.671
Follow-up in years	0.93 ± 0.15	0.95 ± 0.16	0.94 ± 0.15	0.912
Disease duration in years	4.33 ± 2.24	4.10 ± 1.98	3.96 ± 1.80	0.838
MMSE T0	21.61 ± 4.75	20.20 ± 4.68	21.15 ± 3.36	0.370
MMSE T1	18.56 ± 5.03	18.89 ± 5.24	19.94 ± 3.37	0.380
Index of progression	−3.25	−1.39	−1.33	0.021
**Diagnosis N (%)**				0.836
AD	21 (46%)	21 (53%)	26 (57%)	
DLB	1 (2%)	2 (5%)	1 (2%)	
FTD	5 (11%)	2 (5%)	2 (4%)	
MCI	10 (22%)	7 (18%)	6 (13%)	
VAD	9 (20%)	8 (20%)	11 (24%)	
**Therapy N (%)**				0.945
None	13 (28%)	14 (35%)	16 (35%)	
Donepezil	14 (30%)	8 (20%)	11 (24%)	
Rivastigmine	7 (15%)	7 (18%)	6 (13%)	
Donepezil + Memantine	4 (9%)	5 (13%)	6 (13%)	
Other	8 (17%)	6 (15%)	7 (15%)	

*p*-values denote significant difference among all groups on the Kruskal–Wallis test for continuous measures and from the Chi-squared test for categorical measures. Quantitative variables are described using mean ± standard deviation, while qualitative variables are expressed as numbers (percentage). N: number; f: female; m: male; MMSE: mini mental state examination; AD: Alzheimer’s disease; DLB: dementia with Lewy bodies; FTD: frontotemporal dementia; MCI: mild cognitive impairment; VAD: vascular dementia.

## Data Availability

Data supporting the findings of this study are available from the corresponding author, upon reasonable request.
